# A SNP and SSR Based Genetic Map of Asparagus Bean (*Vigna. unguiculata* ssp. *sesquipedialis*) and Comparison with the Broader Species

**DOI:** 10.1371/journal.pone.0015952

**Published:** 2011-01-06

**Authors:** Pei Xu, Xiaohua Wu, Baogen Wang, Yonghua Liu, Jeffery D. Ehlers, Timothy J. Close, Philip A. Roberts, Ndeye-Ndack Diop, Dehui Qin, Tingting Hu, Zhongfu Lu, Guojing Li

**Affiliations:** 1 Institute of Vegetables, Zhejiang Academy of Agricultural Sciences, Hangzhou, People's Republic of China; 2 Department of Botany and Plant Sciences, University of California Riverside, Riverside, California, United States of America; King's College London, United Kingdom

## Abstract

Asparagus bean (*Vigna. unguiculata* ssp. *sesquipedialis*) is a distinctive subspecies of cowpea [*Vigna. unguiculata* (L.) Walp.] that apparently originated in East Asia and is characterized by extremely long and thin pods and an aggressive climbing growth habit. The crop is widely cultivated throughout Asia for the production of immature pods known as ‘long beans’ or ‘asparagus beans’. While the genome of cowpea ssp. *unguiculata* has been characterized recently by high-density genetic mapping and partial sequencing, little is known about the genome of asparagus bean. We report here the first genetic map of asparagus bean based on SNP and SSR markers. The current map consists of 375 loci mapped onto 11 linkage groups (LGs), with 191 loci detected by SNP markers and 184 loci by SSR markers. The overall map length is 745 cM, with an average marker distance of 1.98 cM. There are four high marker-density blocks distributed on three LGs and three regions of segregation distortion (SDRs) identified on two other LGs, two of which co-locate in chromosomal regions syntenic to SDRs in soybean. Synteny between asparagus bean and the model legume *Lotus. japonica* was also established. This work provides the basis for mapping and functional analysis of genes/QTLs of particular interest in asparagus bean, as well as for comparative genomics study of cowpea at the subspecies level.

## Introduction

Cowpea (*Vigna unguiculata* L. Walp.) (2n = 2x = 22), an important annual grain legume, fodder and vegetable crop in many tropical/subtropical regions of the world, is a diploid species belonging to the *Phaseoleae* with an estimated genome size of ∼630 Mb [1]. Phylogenetically, cowpea is close to the warm season legumes such as azuki bean (*Vigna angularis* Willd. Ohwi & Ohashi) and mungbean (*Vigna. radiata* L. Wilczek) of the genus *Vigna*, as well as species of the genus *Phaseolus* and *Glycine*, including the model crop legume soybean *(Glycine max* L. Merr). There are two main cultivated divisions of cowpea worldwide, i.e. the dominant subspecies *unguiculata*, which is widely cultivated in Africa, India, Middle East and the Americas for dry grain and which is a major source of dietary protein for millions of people in developing countries [2], and the subspecies *sesquipedialis*, which is also known as asparagus bean or ‘yard long’ bean in eastern and southern Asia, for production of immature green pods [3]. Asparagus bean is characterized by its striking long (0.5–1 m) pods that are harvested at an immature ‘snap’ stage and its climbing growth habit that is rare in ssp. *unguiculata*, and is considered one of the top ten Asian vegetables. Diverse landrace forms of asparagus bean are found throughout Asia [4], and yet this subspecies is never observed in Africa, suggesting this form of cowpeas arose in Asia from an unknown progenitor form of cowpea long ago.

The extent of genome conservation/diversification between asparagus bean and ssp. *unguiculata* is not well understood; however, the two subspecies are quite distinct morphologically, reflecting the intense selection pressure for adaptation to contrasting environments (hot, drought-prone in Africa compared with higher rainfall and better soil conditions in Asia) and for different uses (primarily dry grain and fodder in Africa compared with tender pods at the immature stage in Asia) [3], [5]. In addition to the differences in pod length and plant architecture, asparagus bean also differs from ssp. *unguiculata* by narrow kidney-shaped seeds. In contrast to the aim of gaining higher yield of seeds with improved nutrition for ssp. *unguiculata*, the main objective in asparagus bean breeding is to obtain more pods per plant and to slow the rate of seed development to prolong the production of attractive immature pods favourable for vegetable use.

Recently, a consensus genetic map of cowpea ssp. *unguiculata* was constructed based on 928 EST-derived SNP markers, providing a solid basis for gene/QTL mapping and genome characterization [6]. However, the cowpea consensus map alone may not be sufficient to investigate traits of particular interest in asparagus bean given that alleles conferring very long pods and climbing habit likely are absent in the ssp. *unguiculata* gene pool [5]. Therefore, a comparable genetic map of asparagus bean is needed to identify genes/QTLs related to specific traits relevant to long bean improvement, as well as to reveal the extent of genome conservation/differentiation between the two subspecies.

Thus far, there has been no genetic map for asparagus bean. In previous work, we developed over 1000 SSR markers from ssp. *unguiculata* DNA sequences, which overall showed more than 98% transferability in asparagus bean [4]. In this paper, we report the construction of a high-density genetic map for asparagus bean employing a combination of the SSR markers and EST-derived SNP markers.

## Results and Discussion

### Polymorphism and segregation of the DNA markers

Amplification of DNA of the mapping parents using 1372 SSR markers gave an overall technical success rate of 92.3% and detected 210 polymorphic loci, providing an average polymorphic rate of 16.5%. Without considering SSRs from Li et al (2001) [7] because of their limited number (46 pairs) that may not be statistically representative, SSRs from gene space sequences (GSS) and bacterial artificial chromosome (BAC) end sequences showed the highest and similar polymorphism rates (20.5% and 20.2%, respectively) compared to EST-SSRs (9.1%) (Table 1). This is within expectation because ESTs are from coding regions of the genome and thus are more conserved [8], [9]. The majority of the polymorphic SSR markers were inherited in a codominant manner and fit the expected 1∶1 segregation; however, segregation of 29 markers (13.8% of total) significantly deviated from this ratio (*P*<0.05). Twelve markers had a minor allele frequency (MAF) value lower than 0.2, demonstrating significant segregation distortion. There were four multi-locus SSR markers (clm0117, clm0126, clm0251, clm0858), meaning they detected more than one polymorphic locus each.

**Table 1:** Summary of DNA markers surveyed.

Type	Code	Number	No. of polymorphic markers	Polymorphism rate (%)	Source
gSSR	VM1-40, VM68-73	46	10	-	Li et al (2001)
	clm0001-0100, clm0201-0330,clm0331-0600,clm1138-1237clm1295-1326	632	121	20.5	Authors’ lab, derived from GSS sequences
	Clm0101-200,clm1038-1137	200	38	20.2	Authors’ lab, derived from BAC end sequences
eSSR	Clm0601-1010,Clm1238-1294	467	41	9.1	Authors’ lab, derived from HarvEST unigenes
	Clm1011-1037	27			Authors’ lab, derived from NCBI ESTs
SNP	1_0001-1_1536	1536	206	14.9%	Muchero et al (2009)

The GoldenGate assay harboring 1378 technically acceptable EST-derived SNPs had 206 polymorphic loci, showing a polymorphism rate of 14%. This value is higher than that of EST-derived SSRs (9.1%), but much lower than those observed in the six ssp. *unguiculata* RIL populations used in Muchero et al. (2009) [6]. Given that the mapping parents used in our study are very distant in geographic origin and are quite different in morphology, this supports the view that the level of genetic diversification in the asparagus bean gene pool is lower than in ssp. *unguiculata*, in agreement with previous genetic diversity studies using AFLP and SSR markers [4], [10]. Eighty-two percent of the polymorphic SNP markers segregated in a 1∶1 manner, while 37 markers exhibited distorted segregation (P<0.05).

### A SSR and SNP based genetic linkage map of asparagus bean

**Figure 1:**
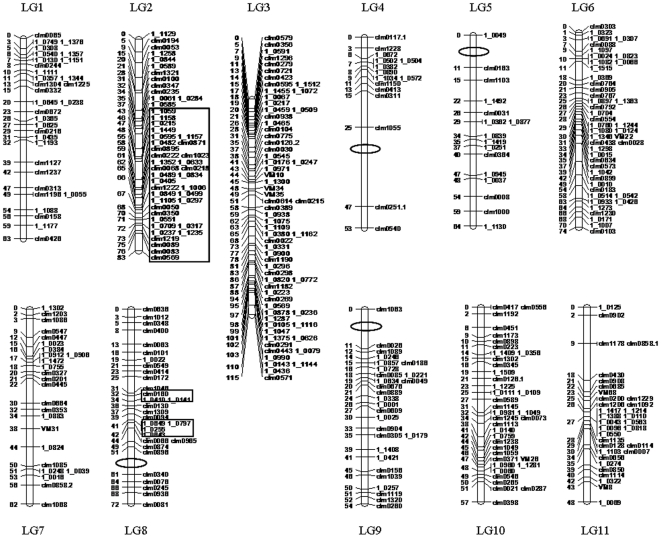
The ‘ZZ’ genetic linkage map of asparagus bean. Numbers to the left of each LG are marker positions (cM). Open ovals indicate gaps greater than 10 cM. Regions defining distorted segregation are boxed.

Three hundred seventy-one markers targeting 375 discrete loci were mapped onto 11 linkage groups (LGs) corresponding to the chromosome pair number of asparagus bean. For consistency, the nomenclature of LG1 to LG11 was assigned according to SNP markers shared with the cowpea consensus map of Muchero et al. (2009) [6]. The current asparagus bean map, referred to as the ‘ZZ’ map (‘ZN016’ x ‘ZJ282’) hereafter, includes 191 EST-derived SNP markers and 184 SSR markers (46 EST-derived, 138 derived from BAC ends or GSS sequences), covering 745 cM of the asparagus bean genome and giving an average marker distance of 1.98 cM (Figure 1, Table S2). The length of individual LGs vary from 48 cM to 115 cM, with the average marker distance per LG ranging from 1.5 cM for the highest to 4.0 cM for the lowest (Table 2). Differential distribution of SNP and SSR markers toward different LGs are evident. For example, LG8 and LG10 are populated predominantly with SSR markers (76% and 65%, respectively), whereas in LG6 64% of the map positions are SNP markers.

**Table 2:** Marker distribution on the 11 linkage groups of asparagus bean.

LG	Number of loci			Length (cM)	Average distance (cM)	No. of gaps >10 cM
	Total	SNP a	SSR			
1	32	19(3)	13	63	2.0	0
2	48	29(1)	19	83	1.7	0
3	65	39(5)	26	115	1.8	0
4	15	7(0)	7	53	3.5	1
5	16	10(0)	6	64	4.0	1
6	45	29(2)	16	74	1.6	0
7	25	12(4)	14	62	2.5	0
8	30	7(0)	23	72	2.4	1
9	28	12(3)	16	54	1.9	1
10	38	13(2)	25	57	1.5	0
11	33	14(1)	19	48	1.5	0
Total	375	191	184	745	1.98	4

a The numbers of SNP loci not included in the cowpea consensus map are given in parentheses.

Four high marker density regions were found on three LGs, one occurring in interstitial regions in LG2, one in interstitial region in LG6 and the remaining two near telomeres of LG3 in opposite directions (Figure. 1, Table S2). There are also four large gaps more than 10 cM distributed on four other LGs, i.e. LG4, LG5, LG8 and LG9. All these gaps are located near the end of the linkage groups. The tendency of gaps toward chromosome ends was also reported in other plant species such as wheat and pearl millet [11], [12], and is considered a reflection of high levels of recombination at distal regions of the chromosomes. The largest gap in LG4 also may indicate a recombination hotspot. Except for the gap in LG5, all other gaps are flanked by SSR markers, thus currently they are not comparable to the cowpea consensus map. The LG5 gap is adjacent to the SNP locus 1_0049 and is not found in the consensus map, demonstrating a low level of SNP polymorphism within this region in the ZZ population. As a next step, additional SNPs and other types of DNA markers such as amplified fragment length polymorphism (AFLP) markers could be applied to fill these gaps.

Three regions of segregation distortion (SDR) were mapped onto two LGs (Figure 1). The LG2 SDR comprises as many as 22 SNP markers and 13 SSR markers, covering a map distance of up to 40 cM and accounting for 58% of the total skewed markers in the map. Ten of these markers, eight being SNPs, co-segregated or tightly linked in a smaller region from the position 65 cM to 72 cM. There are two additional SDRs in LG8, which are adjacent but separated. All the markers in these SDRs exhibited skewed genotypic frequencies toward ‘ZN016’, indicating strong selection of gametes with female alleles at these loci. However, the SDR in LG2 may not affect the calculation of map distance according to theoretical estimates in case only one genetic factor affecting SD is present in a SDR [13]–[15]. The map distance in LG8 might be somewhat deviated from the true values with the assumption that two linked gametophytic factors are likely present in the chromosome [15].

### Comparison of the asparagus bean ZZ map and the cowpea ssp. *unguiculata* consensus map and implications in subspecies diversification

**Figure 2:**
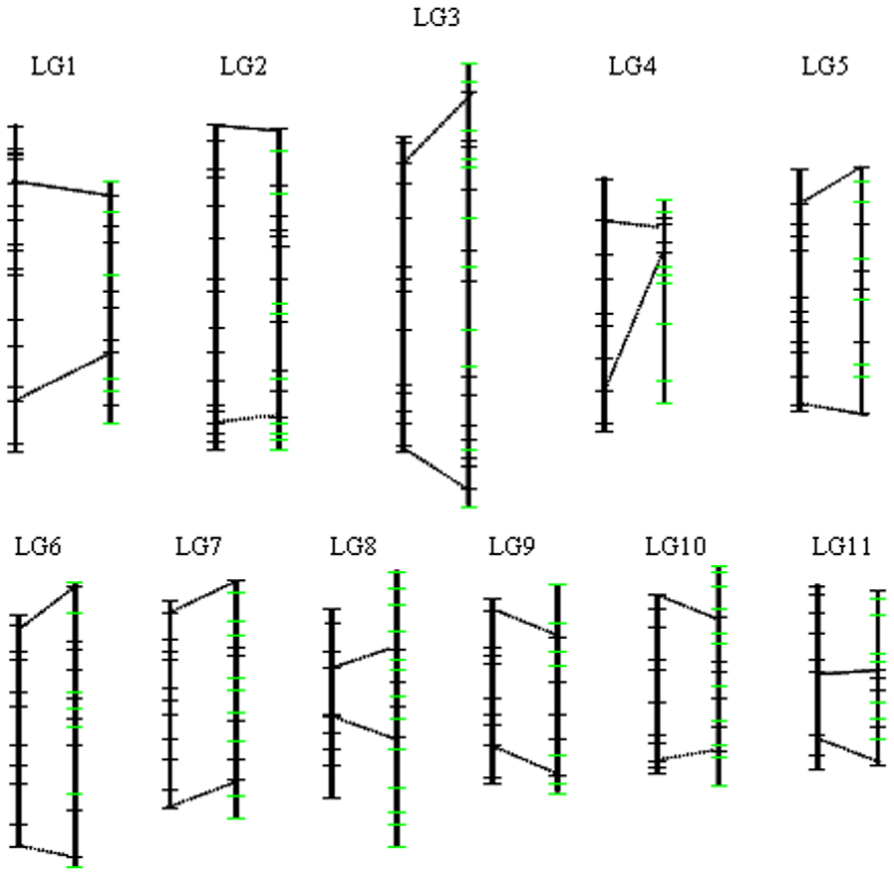
Comparison of the asparagus bean ‘ZZ’ map with the cowpea ssp. *unguiculata* consensus map. For each LG, on the left is the consensus map and on the right is the ZZ map. The length of each vertical line is proportional to the length (cM) of the corresponding LG. Fragments aligned by dashed lines indicate regions covered by SNP markers shared in the two maps. Black and green horizontal bars indicate SNP and SSR markers, respectively. For clarity of presentation, note that not all mapped loci were included in the figure.

The ZZ map, which is 745 cM in length, is only a little longer than the 680 cM consensus map. This map, which is single-population based and thus is not dependent on integration of markers from different populations, may be more accurate in marker order at some positions. The number of 191 mapped SNP markers is much less than that in any of the individual ssp. *unguiculata* mapping populations used in Muchero et al. (2009) [6], where the least number of SNP markers mapped in each population was 288. A significantly lower polymorphism rate between the asparagus bean mapping parents may account for this. However, with the incorporation of SSR markers, the final marker density of the map (1.98 cM) is very close to those of individual ssp. *unguiculata* SNP maps [6]. LG2 and LG3 carry the largest number of mapped loci (Figure 1, Table S2), which is consistent with the consensus map, suggesting high polymorphism of loci on these chromosomes across different populations. LG4 and LG5 have the least mapped loci in ZZ map, while in consensus map LG11 is the least polymorphic. The individual LG lengths are all longer than the corresponding ssp. *unguiculata* consensus map linkage groups, with the exception of LG2 and LG5, which are quite similar between the two maps. The tagging of more chromosomal regions, in particular non-coding regions by SSR markers on the ZZ map are considered the reason for these length variations. We note that some of the SSR markers, together with 21 new SNP markers that were not included in the published consensus map, extend the map coverage in some LGs. For example, as shown in Figure 2, an additional 11 cM region in LG7 beyond the SNP loci 1_0039 and an additional 14 cM region in LG10 above 1_1409 are targeted by SSR markers. The genome coverage portions of LG2, 3, 5, 6 and 9 are very similar to the consensus map according to shared SNP loci, with many additional loci in the same chromosome regions populated by SSR markers, providing additional useful information to further saturate the genetic map. For LG8 and 11, less than half of the chromosomal regions are comparable between the two maps based on common SNP markers, even though the rest of the chromosomal parts, which were mapped by different types of markers, might actually cover the same regions. LG4 is the only one showing major variation between the two maps. Considerably fewer SNP markers are mapped in LG4 of the ZZ map; 1_0572, which is 30cM from 1_1034 in the consensus map, is tightly linked to 1_1034 in ZZ map. However, the total lengths of LG4 in both maps are similar (Figure. 2). The smaller number of markers mapped in LG4 implied that there must be a higher level of conservation in DNA sequences of this chromosome between the mapping parents, suggesting that certain regions of LG4 may be crucial in shaping some subspecies specific traits of asparagus bean. A large gap between two SSR loci (clm1055, clm0251.1) suggests the existence of a recombination hotspot, while the decreased genetic distances among a cluster of SNP markers with respect to ssp. *unguiculata* is indicative of a low rate of recombination. In maize, rates of recombination for specific chromosome segments also are known to vary greatly in different mapping populations [16]–[18]. More work is required to uncover the significance of such structural variation and its functional impact.

A closer look at marker order in the two maps revealed high conservation but also possible differences in genome structure between the two subspecies. Among the shared SNP loci, 90% are in the same order. Of the remaining 10% loci showing inconsistency, 30 (90%) were due to additional crossovers observed in ZZ population thus is not the reflection of genome reorganization. Three markers, i.e., 1_0308 (LG1), 1_1059 (LG2) and 1_1298 (LG6) show slight change in orders between the two maps; however, since these markers are randomly distributed, such changes maybe due to the presence of additional flanking SSR loci or different mapping LOD threshold used. In fact, minor variations of marker order and position are common when comparing genetic maps constructed from different mapping populations [11], [19]. Nevertheless, locations of three markers, 1_0055 (LG1), 1_1116 (LG3) and 1_1034 (LG4) in ZZ map are quite different from those in the consensus map and this couldn't be resolved by removing flanking markers or using different mapping parameters, indicating possible inversion/translocation of the chromosomes. A cluster of nine co-segregated loci in the SDR of LG2 also differed in order from those in the consensus map, which is suggestive of chromosomal rearrangement or meiotic drive and/or gametic selection [15]. Although additional work such as fluorescent in situ hybridization (FISH) is needed to further validate these possible chromosomal changes, extensive marker reorganization including inversion and translocation has been reported between cowpea ssp *unguiculata* and its close relative *Vigna radiata* [20].

### The syntenic relationship between asparagus bean and *L. japonica*

**Figure 3:**
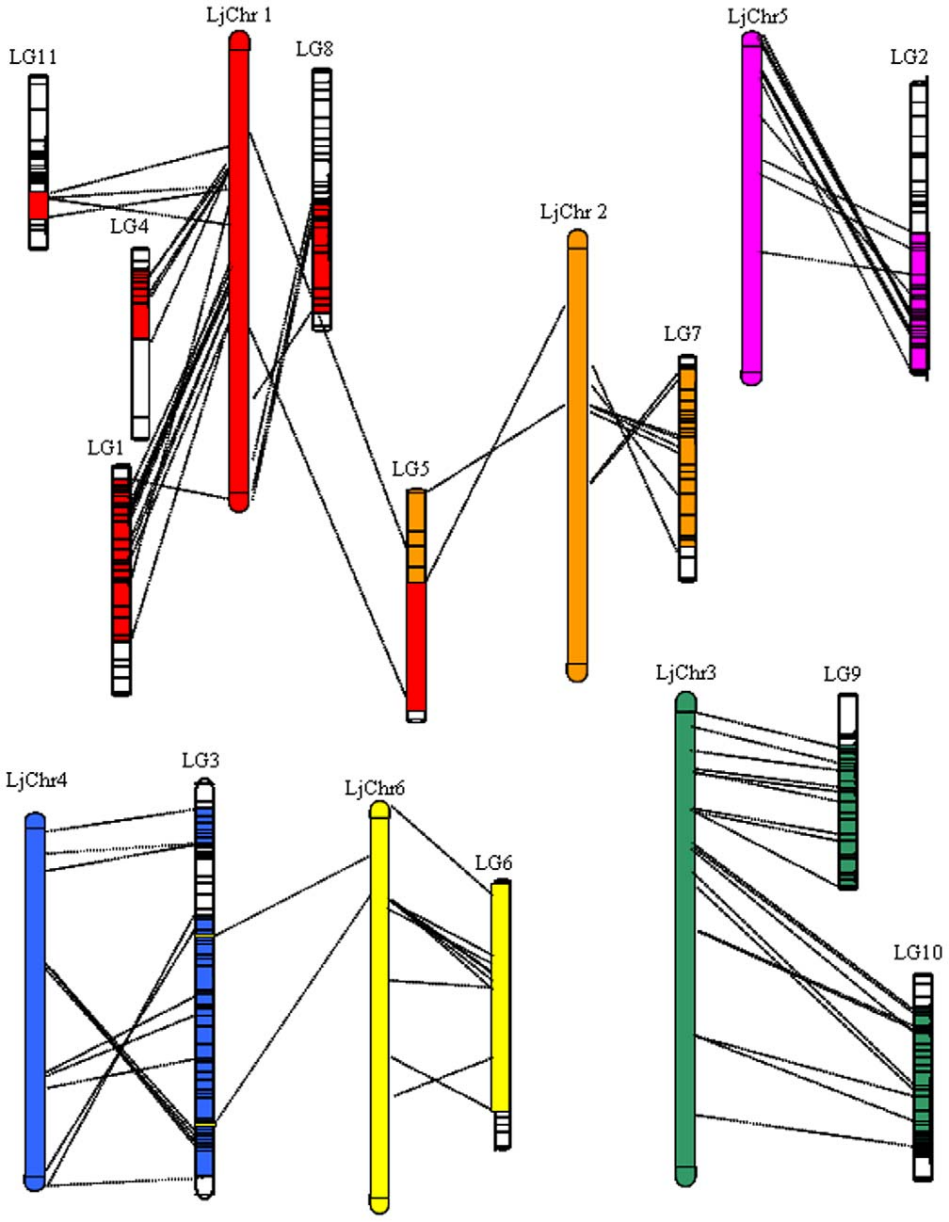
A synteny map between asparagus bean and *Lotus japonica*. Chromosomes of *L. japonica* are marked with LjChr,while asparagus bean linkage groups are marked with LG.

The macrocolinerity between cowpea and the warm-season model legumes soybean and *Medicago truncatula* has been reported [6]; however, the syntenic relationship between cowpea and the more distant cool season model legume *L. japonica* has not yet been established. Here, we show that the genomes of asparagus bean and *L. japonica* are highly syntenic, with macrosynteny punctuated by rearrangements frequently involving translocation and inversion of chromosome arms (Figure 3, Table S3). The ‘chimeric’ nature of synteny also has been observed when comparing cowpea-soybean and medicago-*L. japonicus* [6], [21], which reflects the difference of chromosome numbers between these species. *L. japonicus* chromosome 1 (hereafter referred to as LjChr) exhibits the largest number of matched asparagus bean marker loci and is syntenic to as many as five asparagus bean chromosomes (LG1, 4, 5, 8, 11). LjChr4 and 5 each corresponds to only one asparagus bean chromosome, which may reflect their relative shorter physical lengths [22]. LjChr3 shows the best conservation in order of loci with asparagus bean LG9, implying strongest structural similarity of these regions during species differentiation. Taken together, regions of extensive synteny between asparagus bean and *L. japonicus* provides a framework foundation for further marker and gene identification in asparagus bean through the use of sequence data from *L. japonica* as a genomic model.

### Comparison of SDRs in asparagus bean and soybean

The SDRs in ZZ map were compared with those mapped in soybean genetic map. Yamanaka et al. (2001) reported two SDRs with three or more loci in soybean LG D2 and LG J [23]; Hwang et al. (2009) detected a same SDR in LG I using two different mapping populations [24]. Soybean LG I corresponding to chromosome 20, was syntenic to cowpea LG 2 [6], in which a large SDR exists. When cowpea SNP marker 1_1235, which falls into the LG2 SDR region, was compared using BLAST to the soybean genome sequence, it was found to be syntenic to the locus Glyma20g26670.1, which is adjacent (less than 10 Mb apart) to the mapped LG I SDR. Therefore, it appears that this SDR is conserved in different legumes species, at least in the *Phaseoleae* group. Similarly, SDRs in syntenic regions were also observed in related species of *Poaceae* [11]. Evolutionary distance, mutations and gametophyte genes [15], [25], [26] are considered to be causes of SD. Given that the LG2 SDR is conserved across species, we assume that involvement of gametophytic gene is the most possible causal factor.

### Advantages of integrating SSR and SNP markers for genetic mapping

We present here the first genetic map of asparagus bean that establishes a foundation for genome characterization and molecular breeding of this specialized cowpea subspecies. This map, comprised of two types of DNA markers has some advantages. The successful transfer of high-throughput SNP genotyping platform developed from ssp. *unguiculata* to asparagus bean made the mapping project very efficient and ‘parallel’ to the international ssp. *unguiculata* mapping work, allowing comparison of genetic maps between the subspecies. The development and application of SSR markers, some of which are from EST sequences and others from genomic sequences, complemented the relatively low polymorphism rate of EST-derived SNPs between the mapping parents, and more importantly, enabled us to detect more loci including non-coding regions which are absent in the consensus map. Although SNP assays are becoming more cost-efficient, in some situations SSR markers may be more practical for marker-assisted breeding [8]. One practical path for marker-assisted breeding of asparagus bean would be to use high-throughput SNP genotyping combined with phenotypic information, to identify marker-trait associations, then select nearby SSR markers for repeated marker assisted selection in a breeding program. In the near future, a useful task could be to genotype some of the RIL populations used in constructing the cowpea ssp. *unguiculata* consensus map with the newly available SSR markers and to recalculate the consensus map, by which more informative results could be obtained from comparing the updated maps of the two subspecies.

The ZZ map also provides a significant resource for functional analysis of traits of interest as well as comparative genomics studies. A functional classification of the mapped loci shows a broad range of functional types among these loci. This information is particularly important in gene/QTL mapping of traits such as pod length and climbing habit to help discover candidate genes (Table S2). EST-derived markers linked to target genes could also be used to demarcate orthologous gene regions with model legumes to facilitate development of new markers for fine mapping. The establishment of macrocolinearity among the genomes of cowpea, soybean, *Medicago* [6] and *L. japonica* (this report) would contribute to the detailed analysis of genome evolution of model legumes. With more sequences and mapping data from other important crop legumes such as mungbean [27], chickpea [28] and azuki bean [29] becoming available, soon it will be feasible to draw deeper and clearer insights into genome conservation/diversification among related crop legume species.

## Materials and Methods

### Plant materials

The mapping population, which consists of 114 F_7:8_ recombinant inbred lines (RILs) developed by single-seed descent from a cross between ‘ZN016’, a rustic landrace asparagus bean (*Vigna. unguiculata* ssp. *sesquipedialis*) variety originating from southern China and ‘Zhijiang282’, a commercial asparagus bean cultivar in China, as well as the mapping parents were used in this study.

### DNA extraction

Genomic DNA for SSR analysis was extracted from leaves of two-week-old seedlings using either a modified CTAB method [30] or the DNeasy Plant DNA miniprep kits (Qiagen, Hilden, Germany) according to the manufacturer's procedures. For SNP assay, all the DNA samples were extracted using these kits.

### SNP genotyping and raw data processing

Procedures of the high-throughput SNP genotyping assay and raw data processing employing a 1536-SNP Illumina GoldenGate platform was the same to that as described in Muchero et al (2009) [6], with the exception that SNPs with a MAF<0.3 were retained for mapping in order to include segregation distorted regions (SDRs).

### SSR marker development and PCR

One thousand three hundred and seventy-two microsatellite markers developed in the authors' lab, including 494 EST-SSRs, 632 GSS-SSRs and 200 SSRs derived from BAC end sequences, in addition to 46 SSR markers developed by Li et al (2001) [7] were used. Sequence information of the markers was deposited in Table S1. Refer to Xu et al. (2010) for details of the method for marker development [4].

Polymerase chain reactions (PCR) were carried out in 12.5 µl reactions containing 10 ng of each of the template DNA, 2.5 pmol of each of the primers, 2.5 nmol of each of the dNTPs, 18.6 nmol MgCl_2_, 0.2 U r*Taq* DNA polymerase (Takara, Japan) and 1× PCR buffer supplied together with the enzyme. The PCR cycles were 94°C 3 min, 36 cycles of 94°C 20 s, 48–52°C 30 s depending on primers used, 72°C 40 s, and a 5 min 72°C extension. The PCR products were separated in 8% non-denaturing polyacrylamide gels (Acr:Bis  = 19∶1 or 29∶1) at room temperature in vertical gel apparatus (170×150×1.0 mm) with 1× TBE buffer and visualized by silver staining [31].

### Linkage mapping

Linkage maps were constructed using JoinMap 3.0 (Kyazma, Wageningen, The Netherlands) incorporating the available SNP and SSR markers data. A LOD score of 5.0 with a maximum recombination of 45% was used to assign markers to linkage groups with the exception for LG6 and LG11, where a LOD score of 8.0 and 9.0, respectively, was applied to break spurious linkage according to Muchero et al. (2009). LOD threshold for map calculation was set as 3.0 except for LG2, LG8 and LG4, in which the LOD scores of 2.0, 2.0 and as low as 0.1 were used respectively, in order to allow the inclusion of more distant loci. Other important mapping parameters included: Kosambi's mapping function [32], goodness-of-fit Jump threshold for removal loci  = 5.0, number of added loci after which to perform a ripple  = 1, and third round  = Yes. The segregation fit of each locus to a 1∶1 ratio was examined using the chi-square test. A region with three or more adjacent loci showing significantly skewed segregation (*P*<0.05) was defined as a SDR [33].

### Analysis of synteny

Synteny between asparagus bean and the model legume *L. japonica* was determined by blast N searches against the genome sequence of *L. japonica* (released by Kazuza.org 2009, http://www.plantgdb.org/LjGDB/cgi-bin/blastGDB.pl) using source sequences of mapped asparagus bean SNP and SSR markers as queries. The cut-off value for significance was set as e^-20^ in at least a 120bp overlap.

## Supporting Information

Table S1 **Sequences of SSR primers developed in the authors' lab and used in the current study.**
(XLS)

Table S2 **Position, type and functional annotation of mapped loci.**
(XLS)

Table S3 **Synteny of mapped loci to the** ***L. japonica*** **pseudochromosomes.**
(XLS)
